# Measurement properties of the Health Literacy Questionnaire (HLQ) among older adults who present to the emergency department after a fall: a Rasch analysis

**DOI:** 10.1186/s12913-017-2520-9

**Published:** 2017-08-29

**Authors:** Rebecca L. Morris, Sze-Ee Soh, Keith D. Hill, Rachelle Buchbinder, Judy A. Lowthian, Julie Redfern, Christopher D. Etherton-Beer, Anne-Marie Hill, Richard H. Osborne, Glenn Arendts, Anna L. Barker

**Affiliations:** 1 Department of Epidemiology and Preventive Medicine, Monash University, Melbourne, VIC 3004 Australia; 2 Department of Physiotherapy, Monash University, Melbourne, Australia; 3 School of Physiotherapy and Exercise Science, Curtin University, Perth, Australia; 4 Monash Department of Clinical Epidemiology, Cabrini Institute, Melbourne, Australia; 5 The George Institute for Global Health, University of Sydney, Sydney, Australia; 6 Western Australian Centre for Health & Ageing, University of Western Australia and Royal Perth Hospital, Perth, Australia; 7 Health Systems Improvement Unit, Deakin University Centre for Population Health Research, Geelong, Australia; 8 School of Primary, Aboriginal and Rural Health Care, University of Western Australia, Perth, Australia; 9 Harry Perkins Institute of Medical Research, Perth, Australia

**Keywords:** Older adults, Falls prevention, Health literacy, Measurement properties, Rasch analysis

## Abstract

**Background:**

Health literacy is an important concept associated with participation in preventive health initiatives, such as falls prevention programs. A comprehensive health literacy measurement tool, appropriate for this population, is required. The aim of this study was to evaluate the measurement properties of the Health Literacy Questionnaire (HLQ) in a cohort of older adults who presented to a hospital emergency department (ED) after a fall.

**Methods:**

Older adults who presented to an ED after a fall had their health literacy assessed using the HLQ (*n* = 433). Data were collected as part of a multi-centre randomised controlled trial of a falls prevention program. Measurement properties of the HLQ were assessed using Rasch analysis.

**Results:**

All nine scales of the HLQ were unidimensional, with good internal consistency reliability. No item bias was found for most items (43 of 44). A degree of overall misfit to the Rasch model was evident for six of the nine HLQ scales. The majority of misfit indicated content overlap between some items and does not compromise measurement. A measurement gap was identified for this cohort at mid to high HLQ score.

**Conclusions:**

The HLQ demonstrated good measurement properties in a cohort of older adults who presented to an ED after a fall. The summation of the HLQ items within each scale, providing unbiased information on nine separate areas of health literacy, is supported. Clinicians, researchers and policy makers may have confidence using the HLQ scale scores to gain information about health literacy in older people presenting to the ED after a fall.

**Trial registration:**

This study was registered with the Australian New Zealand Clinical Trials Registry, number ACTRN12614000336684 (27 March 2014).

## Background

Falls represent the main cause of emergency department (ED) presentations for older adults [1]. However, participation in falls prevention activities following presentation to the ED with a fall is suboptimal [2]. Health literacy is an important concept associated with participation in preventive health initiatives [3]. Health literacy is defined as “the cognitive and social skills which determine the motivation and ability of individuals to gain access to, understand and use information in ways which promote and maintain good health” [4].

Adults with sub-optimal health literacy are less likely to participate in preventive health programs, such as falls prevention programs, possibly due to lack of understanding of health information and education provided [5]. Accurate measurement of health literacy prior to commencing a falls prevention program may guide clinicians to adapt provider-patient communication, such as provision of information related to falls risks and their management strategies, to match the patient’s level of health literacy. This may lead to increased participation in falls prevention activities, potentially resulting in improved outcomes for these individuals.

A range of health literacy measurement tools are available. However, most tools do not reflect the multidimensional definition of health literacy, and predominantly focus on reading comprehension, pronunciation and numeracy [6, 7]. The Health Literacy Questionnaire (HLQ) was developed to address the shortcomings of previous tools [8]. The HLQ comprises nine independent scales related to the understanding of, engagement with, and use of health services, from both an individual and organisational perspective.

The measurement properties of the HLQ have been explored in depth using predominantly classical test theory (CTT) approaches [8–11] and qualitative approaches [8, 12]. The HLQ was originally validated using a sample from clinical, home and community care settings in Australia [8]. A highly restrictive 9-factor confirmatory factor analysis (CFA) model fitted satisfactory, with each of the HLQ scales representing nine conceptually distinct areas of health literacy. Subsequent studies evaluating the psychometric properties of the HLQ, including German, Danish, and Slovakian versions, support these findings, with the HLQ demonstrating good model fit and reliability, as well as homogeneity of items within each of the HLQ scales [9–11, 13]. Diverse cohorts were used in these studies representing people with a range of health conditions, receiving a variety of health services. A recent study evaluated the measurement properties of the initial version of the HLQ among people at risk of cardiovascular disease, using Rasch methods [14]. Similar to previous studies, each of the nine HLQ scales were found to measure nine separate constructs of health literacy with good internal consistency. Unclear distinction between some response categories in some HLQ scales was reported and the scales were deemed to be suboptimally targeted in relation to the particular cardiovascular cohort [14]. With the HLQ version used in this study, some disordered thresholds among items in scales 6 to 9 were observed. Kolarcik et al. observed this effect as well and subsequently improved the response options which resulted in lower scores (better targeting), and improved model fit, with no disordered thresholds [13].

Rasch analysis is a modern and unique form of item response theory (IRT) [15]. It involves testing an outcome scale against a mathematical model that operationalises the key principles of good measurement [15–17]. Rasch analysis allows for a unified approach to evaluating several measurement issues, such as unidimensionality, local dependency, response category ordering, item bias and targeting, producing rich data that complements and adds to CTT approaches [15–18]. Rasch analysis is widely accepted as the standard for modern psychometric evaluations of outcome scales [15, 19]. As such, this methodology was deemed to be the most appropriate for this study.

Previous studies provide robust evidence to guide the practical use of the HLQ among a variety of international community and clinical populations. However, the measurement properties of the HLQ have not previously been determined for older adults who have presented to an ED after a fall. The appropriateness of a tool may vary across settings, therefore it is imperative to analyse the HLQ in specific populations prior to applying the tool and interpreting scores [8, 12]. The aim of this study was to use Rasch methods to evaluate the measurement properties of the HLQ in a cohort of older adults who presented to a hospital ED after a fall.

## Methods

### Design

This study was embedded within a multi-centre randomised controlled trial (RCT) of a patient-centred falls prevention program: RESPOND. RESPOND incorporates (1) a home-based assessment; (2) education, goal setting and telephone coaching for management of selected falls risk factors; and (3) healthcare provider communication and community linkage, delivered over 6 months [20]. Ethical approval was obtained from Alfred Health (HREC 439/13) and Royal Perth Hospital (REG 13–128), Monash University Human Research Ethics Committee (HREC) (MUHREC CF13/3869–2013001975) and Curtin University HREC (HR 43/ 2014).

### Participants and setting

Adults aged between 60 and 90 years who presented at two Australian EDs with a fall, and had a planned discharge home within 72 h, were eligible to participate in the RESPOND trial [20]. Exclusion criteria were: current palliative care or terminal illness, requiring hands-on assistance to walk, needing an interpreter, a history of psychoses or social aggression, and cognitive impairment (Mini Mental State Examination (MMSE) <23) [21]. A total of 438 patients were recruited to the RESPOND RCT and completed the HLQ. Of these participants, five withdrew prior to completion of the trial. Data from the remaining 433 participants were used for this study.

### Data collection

Demographic data were collected by members of the research team at the screening and recruitment phase at the participating hospitals, and the initial face-to-face assessment conducted at the participant’s home. The home visit was planned to occur within two weeks of discharge from hospital [20]. The HLQ was self-administered by the participant either prior to or during the home visit.

### The health literacy questionnaire (HLQ)

The HLQ comprises 44 items over nine independent scales, each representing a different element of the overall health literacy construct: (1) Feeling understood and supported by healthcare providers; (2) Having sufficient information to manage my health; (3) Actively managing my health; (4) Social support for health; (5) Appraisal of health information; (6) Ability to actively engage with healthcare providers; (7) Navigating the healthcare system; (8) Ability to find good health information; and (9) Understanding health information well enough to know what to do. There are four to six items in each scale. Depending upon the purpose of inquiry, the full instrument or selected scales can be used. The first five scales comprise items that ask the respondents to indicate their level of agreement on one of four response options (strongly disagree to strongly agree). The remaining scales (6–9) represent scales of self-reported capability and items within these scales are scored on one of five response options (cannot do; very difficult; quite difficult; quite easy; very easy). The full HLQ provides nine individual scores based on an average of the items within each of the nine scales. There is no overall total score for the HLQ as that could potentially mask individual needs in specific health literacy domains [22].

### Other measures

Socio-economic status (SES) was measured using The Index of Relative Socio-economic Advantage and Disadvantage (IRSAD) [23], a reliable and robust approach to assessing socio-economic status [24]. Data are based on participant postcodes and take into consideration socio-economic factors such as income, education, employment, occupation and housing [23]. The 20% most advantaged, according to their IRSAD score, were considered to be a relatively high socio-economic group for the purpose of this study. The remaining participants were combined into a second group representing lower socio-economic status.

Whether or not participants have private health insurance or live alone were self-report questions answered yes/no at the time of the initial face-to-face assessment. Falls risk status was measured at the face-to-face interview using a reliable assessment tool: the Falls Risk for Older People – Community setting (FROP-Com) [25]. A FROP-Com score > 18 represented high falls risk [25].

### Analyses

Descriptive statistics were used to profile the cohort using SPSS v22.0 (IBM Corporation, Armonk, New York). Rasch analysis was conducted using the partial credit model, as this allows the thresholds to vary for each of the individual items [26], using RUMM2030 software (RUMM Laboratory Pty Ltd., Perth, Australia). In order to determine whether the HLQ scales fit the Rasch model, response patterns to HLQ items were evaluated against the model’s expectations [15]. Three statistics were considered to determine the degree of fit for each HLQ scale: overall fit; individual person fit; and individual item fit [15]. Adequate overall fit of the HLQ to the Rasch model was indicated by a non-significant Bonferroni adjusted Chi-square probability value [27] (*p* ≥ 0.0125 for four item scales (1 and 2); *p* ≥ 0.01 for five item scales (3, 4, 5, 6, 8 and 9); *p* ≥ 0.0083 for the six item scale (7)). Satisfactory overall item and individual fit for each scale was determined by a fit residual standard deviation (SD) value of ≤1.5 [27].

Individual items were further analysed to determine whether or not each of the four to six items comprising the nine HLQ scales fit the Rasch model requirements. Individual item fit was indicated by two statistics: fit residual values; and Chi-square probability values [16]. Item fit residual values −2.5 to 2.5 indicated adequate fit [28]. Above this range (underfit) suggests deviation from the model, below (overfit) suggests that some items in the scale are similar to each other [26]. Consistent with overall fit, a non-significant Bonferroni adjusted Chi-square probability value (*p* > 0.0125 for scales 1 and 2; *p* > 0.01 for scales 3, 4, 5, 6, 8, and 9; and *p* > 0.0083 for scale 7) indicated adequate item fit [28].

In addition to model fit the following measurement properties were analysed: unidimensionality; internal consistency reliability; response format; item bias; and targeting. Measurement properties analysed, their definitions, statistical tests used and criteria for assessment are summarised in Table 1.

**Table 1:** Measurement properties analysed and criteria for assessment

Measurement property	Definition	Statistical test and ideal values
Unidimensionality	Whether or not each of the nine HLQ scales measures a single health literacy construct [18].	% of significant t-tests from the Principal Components Analysis (PCA) of the standardised residuals <5% indicates unidimensionality. Where >5% significant t-tests, if lower bounds of CI < 0.05, unidimensionality is supported [16, 33].
	Local independence is an element of unidimensionality. This occurs where the response to one item is not dependent on the response to another item [18, 26].	Person-item residual correlation value <0.2 indicates local independence [34].
Internal consistency reliability	The degree to which items in each scale measure the same construct [16].	Person Separation Index (PSI) > 0.7 indicates good internal consistency reliability [15, 28, 34].
Response format	Whether or not participants are able to consistently choose a response category appropriate for their level of health literacy. The point between two response categories (such as strongly agree and agree) where either response is equally probable is known as a ‘threshold’ [28].	The absence of disordered thresholds on the category probability curve graphs indicates appropriate response format [34].
Item bias	Whether or not different subgroups within the sample respond differently to an item, despite having equal levels of health literacy [16, 18]. This is measured using differential item functioning (DIF). Item bias for gender (male or female) and age group (60–75 and 76–90) were analysed.	A Bonferroni adjusted *p* value for significance was used for the DIF analysis [16]: *p* > 0.006 for 4 item scales (1 and 2); *p* > 0.005 for five item scales (3, 4, 5, 6, 8 and 9); and *p* > 0.004 for the six item scale (7) indicating no item bias.
Targeting	The degree to which the HLQ was appropriately targeted to the RESPOND cohort [16].	Targeting was evaluated through analysis of person-item distribution graphs [35]. The mean person location should approximate zero for a well targeted tool [16]. A positive person mean suggests that on the whole respondents found the scales easy to endorse. A negative person mean suggests that respondents found the scales difficult to endorse. A well targeted scale should see items spanning across the full range of individual person scores.

## Results

### Participant characteristics

The mean age of participants was 73 years, 55% were female, and 42% of participants lived alone. Most had private health insurance (61%), and most were of high SES (62%). Approximately one third (34%) were classified as being at high risk of falls. Participant characteristics and HLQ scores are presented in Table 2.

**Table 2:** Participant characteristics

Gender	
Female, *n* (%)	237 (54.7%)
Age	
Mean age (yrs)	72.5
60–75, *n* (%)	271 (62.6%)
76–90, *n* (%)	162 (37.4%)
Private health insurance	
Yes, *n* (%)	264 (61%)
Lives alone	
Yes, *n* (%)	180 (41.6%)
High falls risk	
Yes, *n* (%)	148 (34.2%)
Socio-economic status (IRSAD)	
High socio-economic status, *n* (%)	267 (61.7%)
HLQ score, mean (SD)	
*Section one: scales of agreement. Range 1 (lowest) to 4 (highest)*	
1) Feeling understood and supported by healthcare providers	3.24 (0.28)
2) Having sufficient information to manage my health	3.00 (0.34)
3) Actively managing my health	2.96 (0.33)
4) Social support for health	3.10 (0.41)
5) Appraisal of health information	2.76 (0.44)
*Section two: scales of capabilities. Range 1 (lowest) to 5 (highest)*	
6) Ability to actively engage with healthcare providers	4.15 (0.31)
7) Navigating the healthcare system	4.01 (0.40)
8) Ability to find good health information	3.91 (0.43)
9) Understanding health information well enough to know what to do	4.15 (0.38)

### Rasch analysis

Three of the nine scales: (5) Appraisal of health information; (8) Ability to find good health information; and (9) Understanding health information well enough to know what to do -demonstrated adequate overall fit to the Rasch model as indicated by a non-significant Bonferroni adjusted Chi-square probability value (*p* = 0.33; *p* = 0.02; *p* = 0.05 respectively) (Table 3). The remaining scales demonstrated some degree of misfit between the data and the Rasch model (scales 1 and 2 *p* < 0.0125; scales 3, 4 and 6 *p* < 0.01; scale 7 *p* < 0.0083). The majority of item misfit, as determined by a negative item fit residual value below −2.5 (17 items), suggested overfit (Table 4). A further seven items (one item from each of scales 1, 2, 3, 4, 6, 7, and 8) demonstrated underfit with a Chi-square probability below the adjusted alpha value (scale 1 and 2 *p* < 0.0125; scales 3, 4, 6, and 8 *p* < 0.01; and scale 7 *p <* 0.0083) (Table 4).

**Table 3:** Model fit statistics for HLQ scales

Rasch component	Overall model fit	Item fit Mean (SD)	Person fit Mean (SD)	Internal consistency reliability (PSI)	Unidimensionality (% of significant t tests). CI shown where % of significant t tests >5%
*Section one: scales of agreement (four response categories)*					
1) Feeling understood and supported by healthcare providers	χ^2^ = 27.80	−2.26	−0.92	0.78	2.31%
	***p*** **< 0.0125**	(0.94)	(1.16)		
2) Having sufficient information to manage my health	*χ* ^2^ = 58.10	−2.20	−0.81	0.75	3.70%
	***p*** **< 0.0125**	**(2.51)**	(1.13)		
3) Actively managing my health	*χ* ^*2*^ = 43.21	−2.28	−1.235	0.73	6.47%
	***p*** **< 0.01**	**(1.99)**	**(1.81)**		CI:0.04–0.09
4) Social support for health	*χ* ^*2*^ = 55.62	−0.77	−0.86	0.72	4.85%
	***p*** **< 0.01**	**(2.51)**	**(1.69)**		
5) Appraisal of health information	*χ* ^*2*^ = 22.16	−0.80	−0.81	0.79	6.00%
	*p* = 0.33	**(1.55)**	**(1.60)**		CI:0.04–0.08
*Section two: scales of capabilities (five response categories)*					
6) Ability to actively engage with healthcare providers	*χ* ^*2*^ = 27.77	−2.20	−1.00	0.74	3.46%
	***p*** **< 0.01**	(1.17)	(1.42)		
7) Navigating the healthcare system	*χ* ^*2*^ = 46.64	−2.00	−0.86	0.82	4.16%
	***p*** **< 0.0083**	**(2.43)**	(1.34)		
8) Ability to find good health information	*χ* ^*2*^ = 28.65	−1.36	−0.95	0.77	4.39%
	*p* **=** 0.02	(0.80)	(1.42)		
9) Understanding health information well enough to know what to do	*χ* ^*2*^ = 18.58	−2.03	−0.94	0.72	5.31%
	*p* **=** 0.05	(1.26)	(1.40)		CI:0.03–0.07

*SD* standard deviation, *PSI* person separation index, *CI* confidence interval

Statistics beyond the pre-specified ideal values are noted in bold

**Table 4:** Individual item fit statistics

HLQ scale	HLQ item	Location	SE	Item fit residual	Chi-square	Bonferroni adjusted Chi-square probability
*Section one: scales of agreement (four response categories)*						
1) Feeling understood and supported by healthcare providers	I have at least one healthcare provider who …	0.20	0.10	**−2.73**	3.60	0.17
	I have at least one healthcare provider I can …	0.04	0.11	**−2.85**	2.46	0.29
	I have the healthcare providers I need …	0.40	0.12	−0.87	21.21	**<0.0125**
	I can rely on at least one …	−0.63	0.12	**−2.61**	0.53	0.76
2) Having sufficient information to manage my health	I feel I have good information about health …	−0.56	0.10	1.50	35.02	**<0.0125**
	I have enough information to help me deal …	−0.14	0.10	**−3.98**	6.67	0.08
	I am sure I have all the information I need to …	0.42	0.10	**−3.45**	8.55	0.04
	I have all the information I need to …	0.28	0.09	**−2.86**	7.86	0.05
3) Actively managing my health	I spend quite a lot of time actively managing …	0.48	0.09	0.461	20.54	**<0.01**
	I make plans for what I need to do to be …	0.19	0.10	−1.384	3.63	0.30
	Despite other things in my life, I make time …	0.08	0.10	**−4.56**	5.44	0.14
	I sent my own goals about health and fitness	−0.29	0.11	−2.09	3.57	0.31
	There are things that I do regularly …	−0.46	0.10	**−3.80**	10.03	0.02
4) Social support for health	I can get access to several people who …	−0.25	0.09	0.70	7.08	0.13
	When I feel ill, the people around me really …	0.27	0.09	0.22	7.42	0.12
	If I need help, I have plenty of people I …	−0.09	0.09	**−2.91**	11.27	0.02
	I have at least one person …	0.60	0.08	2.02	10.69	0.03
	I have strong support from …	−0.52	0.09	**−3.87**	19.17	**<0.01**
5) Appraisal of health information	I compare health information from different …	−0.02	0.09	−0.15	0.84	0.93
	When I see new information about health, I …	0.50	0.09	−1.864	2.49	0.65
	I always compare health information from …	0.36	0.09	**−2.88**	8.58	0.07
	I know how to find out if the health …	−0.56	0.10	−0.09	3.86	0.42
	I ask healthcare providers about the quality …	−0.28	0.09	0.98	6.38	0.17
*Section two: scales of capabilities (five response categories)*						
6) Ability to actively engage with healthcare providers	Make sure that healthcare providers understand …	−0.73	0.11	−1.75	6.19	0.05
	Feel able to discuss your health concerns with a …	−0.33	0.11	−1.28	10.84	**<0.01**
	Have good discussion about your health …	0.01	0.10	**−3.61**	5.09	0.08
	Discuss things with healthcare providers …	0.37	0.10	**−3.27**	1.94	0.38
	Ask healthcare providers questions to get …	0.68	0.10	−1.06	3.71	0.16
7) Navigating the healthcare system	Find the right healthcare	−0.03	0.09	−1.44	1.58	0.45
	Get to see the healthcare providers I need to	−0.29	0.09	−1.06	8.55	0.01
	Decide which healthcare provider you need …	−0.43	0.09	**−3.27**	7.35	0.03
	Make sure you find the right place to get …	−0.34	0.09	**−6.19**	4.98	0.08
	Find out what healthcare services you are …	0.68	0.08	0.82	8.53	0.01
	Work out what is the best care for you	0.41	0.09	−0.87	15.66	**<0.0083**
8) Ability to find good health information	Find information about your health problems	−0.25	0.09	−1.70	1.91	0.59
	Find health information from several …	0.48	0.07	−1.26	3.72	0.29
	Get information about health so you are …	0.15	0.08	**−2.51**	4.62	0.20
	Get health information in words you …	−0.86	0.09	−0.44	12.92	**<0.01**
	Get health information by yourself	0.48	0.07	−0.87	5.49	0.14
9) Understanding health information well enough to know what to do	Confidently fill medical forms in the correct …	0.25	0.07	−1.70	3.84	0.15
	Accurately follow the instructions from …	−0.35	0.09	−0.37	2.33	0.31
	Read and understand written health …	0.23	0.08	**−3.87**	7.06	0.03
	Read and understand all the information on …	0.15	0.08	−2.35	3.45	0.18
	Understand what healthcare providers are …	−0.28	0.10	−1.87	1.90	0.39

*SE* standard error

Statistics beyond the pre-specified range are noted in bold

Items are truncated. Full items are available from the tool developers

Good person fit was demonstrated for the majority of the scales (1, 2, 6, 7, 8, and 9) with a person fit residual SD < 1.5 indicating that overall people responded to items as expected. Minor person misfit was shown across three of the nine scales: (3) Actively managing my health; (4) Social support for health; and (5) Appraisal of health information, with a person fit residual SD >1.5 (Table 3). This suggest that some people responded in an unusual way to some items in these scales.

Unidimensionality is a critical property of good measurement and a prerequisite to the summation of items within a scale [15, 29]. Unidimensionality was demonstrated for all nine scales (Table 3) as determined by <5% significant *t*-tests (scales 1, 2, 4, 6, 7, and 8) or a 95% confidence interval (CI) including 5% where >5% significant *t*-tests were evident: scale (3) CI:0.04–0.09; scale (5) CI:0.04–0.08; and scale (9) CI:0.03–0.07. Local independence further supports the concept of unidimensionality [29]. All nine scales demonstrated local independence with between-item residual correlations matrix values <0.2. The Person Separation Index (PSI) for all scales was >0.7 indicating good internal consistency reliability.

**Fig. 1:**
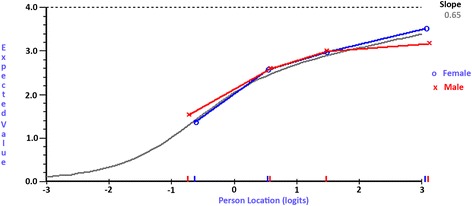
Item characteristic curve depicting DIF. Item characteristic curve for ‘Get health information by yourself’ from scale (8) Ability to find good health information, indicating item bias between males and females

No item bias was evident for the majority of the HLQ items (43 out of 44), demonstrating that people with the same level of health literacy consistently responded to items in the same way, regardless of their gender or age group. Only one item: ‘Get health information by yourself’ from scale (8) Ability to find good health information, demonstrated item bias for gender as indicated by a probability value below the Bonferroni adjusted probability value (*p* < 0.005). This means that males and females responded differently to each other despite having the same level of health literacy (non-uniform DIF) [16] (Fig. 1).

Overall, the response format was found to be satisfactory for the ‘strongly disagree to strongly agree’ scales (scale 1 to 5) as indicated by the absence of disordered thresholds. Mild disordering was evident in scale (4) Social support for health, for the following item: ‘I have at least one person who can come to medical appointments with me’. Disordered thresholds predominantly occurred among the capability response categories (cannot do to very easy) for the following items: ‘discuss things with healthcare providers…’ and ‘Ask healthcare providers questions to get…’ from scale (6) Ability to actively engage with healthcare providers; ‘Find out what healthcare services you are…’ from scale (7) Navigating the healthcare system; ‘Find health information from several…’, Get information about health so you are…’, and ‘Get health information by yourself’ from scale (8) Ability to find good health information; and all items in scale (9) Understanding health information well enough to know what to do. On inspection of the category probability curves, the main issue participants had was choosing between ‘very difficult’ and ‘quite difficult’. The HLQ authors, however, recently changed the capability response options (scales 6–9) to include elements of frequency as well as difficulty, and this was found to be better than the original options [13].

**Fig. 2:**
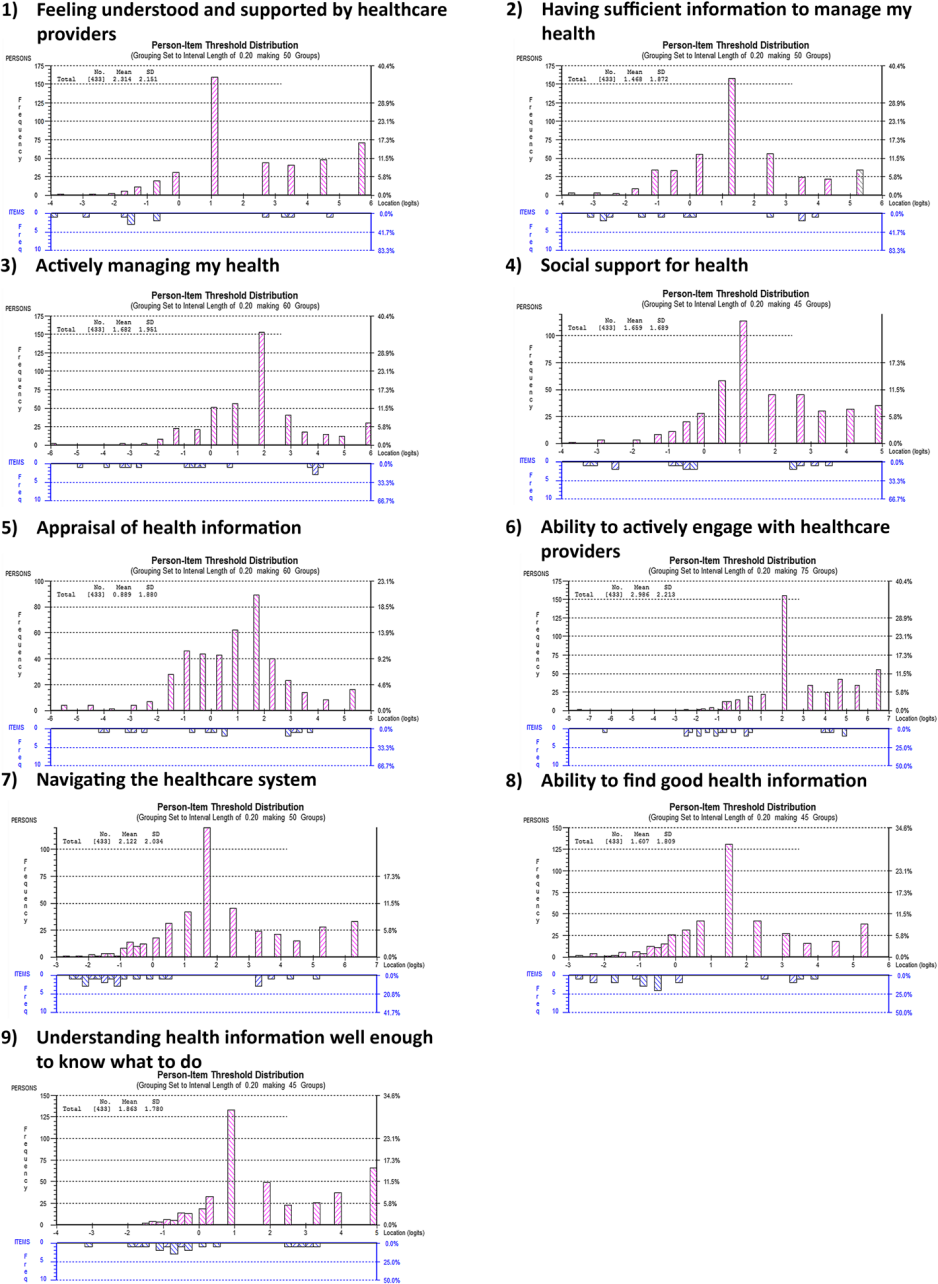
Person-item threshold distribution graphs depicting targeting for the nine HLQ scales. A positive mean person location for all nine scales (0.89–2.99) suggests that participants found some of the items easy to endorse. A measurement gap is evident for all nine HLQ scales - no items match participants’ health literacy level at approximately the one to two logit point (mid to high HLQ score) despite a number of participants at this ability level for each scale

In terms of targeting, a positive mean person location for all nine scales (0.89–2.99) suggested that participants found some of the items easy to endorse. Person-item distribution graphs plot item difficulty and the person’s level of health literacy along a common measure: logits. A logit is the unit of measurement that results when the Rasch model is used to transform raw scores from ordinal data to log odds ratios on a common scale [26]. The value of zero is allocated to the mean of the item difficulty [16, 26]. There should be an even spread of HLQ items across the range of participants’ health literacy levels. On inspection of these graphs there were no items matching participants’ level of health literacy at approximately the one to two logit point (mid to high HLQ score) despite a number of participants at this ability level for each scale (Fig. 2).

## Discussion

This is the first study to assess the measurement properties of the HLQ among a cohort of older people who have presented to an ED after a fall. Health literacy is an important factor associated with participation in preventive health programs, such as falls prevention initiatives. Overall, the HLQ demonstrated good measurement properties. The summation of the HLQ items within each scale to provide scale summary scores, with each scale representing one distinct component of health literacy, is supported. This finding is consistent with previous validation studies of the HLQ [8–11, 14]. This indicates that each HLQ scale measures what it purports to measure, and nothing more, providing detailed information on nine separate areas of health literacy.

Absence of item bias is considered a fundamental principle of good measurement [15, 18]. It is important that items work consistently for individuals across different sub-groups, particularly if different demographic groups are to be compared [18]. Almost all the items (43 of 44) did not demonstrate item bias for the covariates assessed, with minor bias demonstrated for only one item. This suggests that un-biased estimates of health literacy across gender and age groups can be obtained from the HLQ. This finding further supports previous studies that found both the English and Slovakian versions of the HLQ to be invariant across a number of key demographic groups [9, 13].

In this study, the majority of misfit suggests that the set of items within some scales may have overlapping content (overfit). Overfit does not compromise good measurement [26]. A strong rationale for including the items is provided in the development of the tool. Multiple structured processes were undertaken to develop the HLQ items, guided by the revised Bloom’s taxonomy, to generate items of various difficulty. Detailed psychometric analyses were used to test and refine the items, leading to removal or re-wording of poorly performing items [8]. Given the rigorous development process of the HLQ, deletion of misfitting items is not recommended. Doing so may compromise construct coverage and result in loss of some of the tool’s important items [26]. Overall misfit to the Rasch model should be treated with caution. While Chi-square probability values are recommended to determine fit, these values are sensitive to sample size [30]. Given a sufficiently large sample size (*n* = 433 in this study), even small deviations from model fit will be statistically significant [30].

All nine HLQ scales were found to be inadequately targeted for this sample, which is consistent with findings from Richtering et al. [14]. It is important to note that the RESPOND cohort were not representative of the general population in several ways. Firstly, the cohort consisted of participants who were taking part in a clinical trial. Those who volunteer to participate in research projects may have levels of education, motivation and engagement that differ from those who decline to participate. Secondly, due to the exclusion criteria necessary for the purpose of the RCT, the sample was underrepresented for certain subgroups known to have lower levels of health literacy. For example, those born overseas or who speak languages other than English at home, those with lower education, no private health insurance, multiple chronic conditions, and women have been found to have lower health literacy on some HLQ scales [31]. The RESPOND cohort had higher HLQ scores in seven of the nine HLQ scales (scales 1, 2, 4, 6, 7, 8, and 9), and similar levels of health literacy in two scales (3 and 5), when compared to a sample representing a diverse range of socio-economic and geographical characteristics [31]. This may explain why the RESPOND cohort appeared to find some HLQ items easy to endorse. The measurement gap identified has implications for measurement precision, which decreases at the level corresponding with this gap [32]. This means that a large change in health literacy is necessary in order to elicit a change in mid to high HLQ score for the RESPOND cohort.

The main strength of this study is that the sample was from a multi-centre trial, encompassing two geographically diverse areas of Australia. In terms of limitations, the sample size may have contributed to the significant Chi-square probability values [30]. A further limitation was that the sample was under representative of a number of socio-economic groups, limiting generalisability of the results to the broader population of older adults who present to an ED after a fall.

## Conclusions

The current study builds on previously established strong measurement properties of the HLQ and adds new knowledge specific to a population of older people who have presented to an ED after a fall. Overall, the HLQ was found to have good measurement properties among this cohort. The HLQ may be used to tailor falls prevention initiatives to allow for program components programs, such as provision of education, support and community linkage, to be delivered in a manner appropriate for individual health literacy ability. This may increase participation in falls prevention activities, potentially resulting in better health outcomes for these patients.

## Abbreviations

CFA: Confirmatory factor analysis
CI: Confidence interval
CTT: Classical test theory
ED: Emergency department
FROP-Com: Falls Risk for Older People – Community setting
HLQ: Health Literacy Questionnaire
IRSAD: Index of Relative Socio-economic Advantage and Disadvantage
IRT: Item response theory
MMSE: Mini Mental State Examination
PCA: Principal Components Analysis
PSI: Person Separation Index
RCT: Randomised controlled trial
SD: Standard deviation
SE: Standard error
SES: Socio-economic status
